# Imlifidase for hematologists: Creating a therapeutic window in the presence of neutralizing alloantibodies

**DOI:** 10.1002/hem3.37

**Published:** 2024-01-28

**Authors:** Roger E. G. Schutgens

**Affiliations:** ^1^ Center of Benign Haematology, Thrombosis and Haemostasis, Van Creveldkliniek, University Medical Centre Utrecht University Utrecht Utrecht The Netherlands

Bacterial enzymes that specifically break down IgG antibodies have gained increased interest recently, as they have the potential to have a significant impact on the treatment of allo‐ and autoimmune disorders. The Immunoglobulin G‐degrading enzyme of *Streptococcus pyogenes* (IdeS) is normally secreted by *S. pyogenes* during infection. IdeS is now known as imlifidase, and clinical trials have been published on HLA‐sensitized candidates for renal transplantation and anti‐glomerular basement membrane disease.^1^, ^2^ Imlifidase has been studied in a range of hematologic disorders as well. A short overview is given here.

## WORKING MECHANISM OF IMLIFIDASE

**Figure 1:**
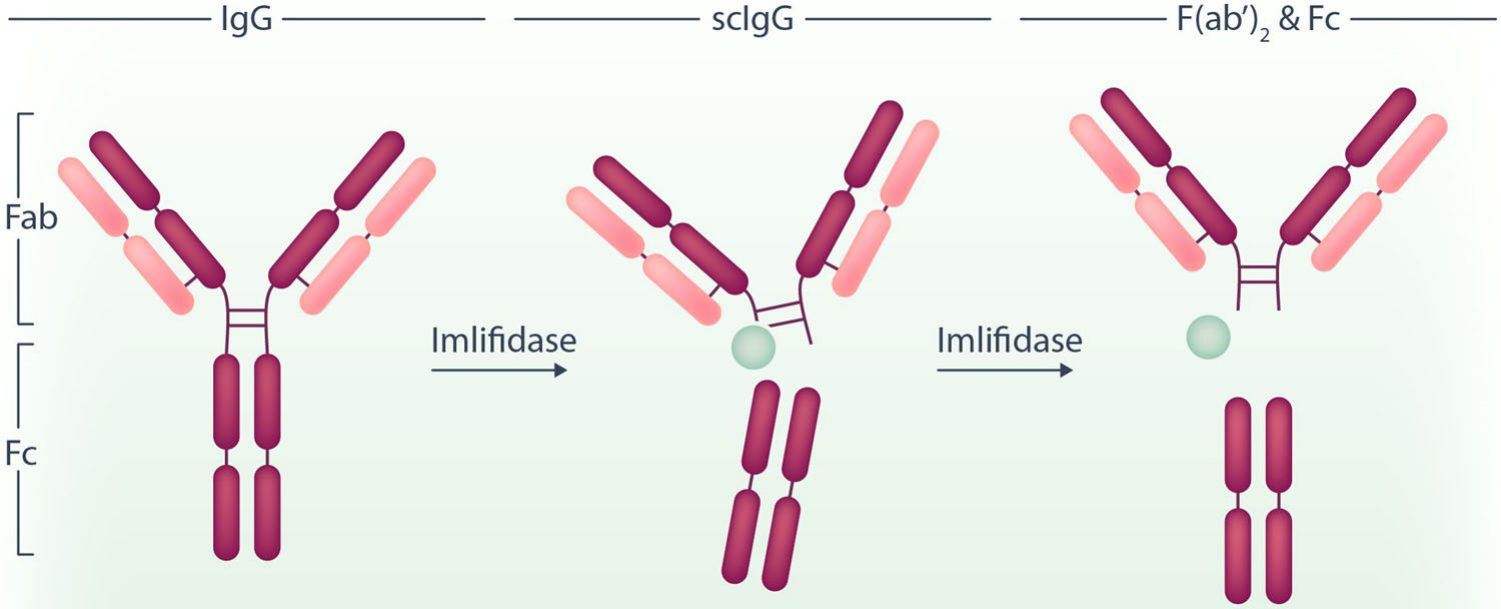
Working mechanism of imlifidase. Imlifidase cleaves the disulfide bonds between the IgG heavy and light chains, resulting in one F(ab')2 and two monomeric Fc fragments.

An IgG molecule consists of two heavy and two light chains held together by disulfide bonds. Imlifidase has a unique specificity for IgG and cleaves IgG in the heavy chains, which generates one F(ab՛)2 and two monomeric Fc fragments^3^ (Figure 1). This leads to a rapid degradation of all circulating IgG within 6 h, with antibody levels returning to baseline around Day 14 postinfusion.^4^ Imlifidase itself will be cleared from the circulation within 24 h. The theoretical concept for imlifidase is that antibody‐driven disorders could temporarily be mitigated.

## THROMBOTIC THROMBOCYTOPENIC PURPURA (TTP)

Immune TTP (iTTP) is an autoimmune disorder where autoantibodies against ADAMTS13 prevent cleavage of long von Willebrand factor molecules, leading to thrombotic microangiopathy. Two cases of iTTP in clinical remission with persistent ADAMTS13 activity <5% and anti‐ADAMTS13 IgG antibodies received a single dose of imlifidase.^5^ Both patients were extensively treated with immunosuppressive agents before, including steroids, rituximab, bortezomib, and mycophenolate mofetil. Although a notable reduction in the levels of specific anti‐ADAMTS13 antibodies was observed, no recovery in ADAMTS13 activity was seen, probably due to residual F(ab')2 antibody binding to ADAMTS13. Both patients experienced an acute serum sickness reaction. It is unclear whether these 2 cases are representative of the general acute iTTP population. Currently, there are no initiatives for a subsequent clinical trial in iTTP.

## HEMOPHILIA

One of the most challenging problems within the treatment of hemophilia nowadays is the formation of antidrug antibodies; this is especially true for treatment with FVIII products. In addition, formation of autoantibodies to endogenous FVIII is the hallmark of acquired hemophilia A. It is therefore of no surprise that imlifidase is being explored in this field as well.

Earlier this year, imlifidase was shown to eliminate FVIII inhibitors in vitro and in a model of inhibitor‐positive hemophilia A mice.^6^ Imlifidase cleaved anti‐FVIII plasma IgG from patients with congenital and acquired hemophilia A in vitro. In mice passively immunized with recombinant human anti‐FVIII IgG, imlifidase restored the hemostatic efficacy of FVIII. These experiments show the proof of concept of transient removal of FVIII inhibitors by imlifidase, creating a therapeutic window during which FVIII supplementation can be given efficiently in inhibitor patients.

Many patients with hemophilia are being treated with the bispecific antibody emicizumab, both patients with and without FVIII inhibitors. The abovementioned concept of creating a therapeutic window in inhibitor patients is an extra challenge in patients treated with emicizumab, as emicizumab itself could be eliminated by imlifidase. This was investigated by the same group recently in a hemophilia mice model.^7^ The levels of emicizumab were reduced by 71% 72 h after administering a set of two injections of equimolar amounts of imlifidase; this decrease was enhanced to 90% when a 10‐fold excess of imlifidase was infused. Circulating F(ab՛)2 fragments of emicizumab were undetectable after 18 hours. More importantly, the estimated procoagulant activity of emicizumab confirmed the major elimination of the functionally active molecule, as peak reduction in procoagulant was 88% and circulating emicizumab concentrations decreased to 3.6 µg/mL. These experiments indicate that imlifidase eliminates both neutralizing anti‐FVIII antibodies and functional activity of emicizumab in hemophilia A. This might create the therapeutic window for efficient FVIII supplementation, while the short T1/2 of imlifidase allows for retreatment with emicizumab very shortly. Although these reports are very encouraging, there are as yet no clinical data on the use of imlifidase in hemophilia patients.

In the current treatment landscape for hemophilia, gene therapy is emerging using adeno‐associated virus (AAV) vectors. The presence of pre‐existing anti‐AAV antibodies precludes this form of therapy in some patients. In mice and nonhuman primates, imlifidase was able to decrease anti‐AAV antibodies and enable efficient liver gene transfer.^8^ Data in humans confirmed the ability of imlifidase to reduce these antibodies in vitro.

## PLATELET REFRACTORINESS

Alloimmune platelet refractoriness has a significant impact on the management of leukemia or hematopoietic stem cell transplantation. In a case report, a 43‐year‐old woman was described with extensive sensitization, profound thrombocytopenia, and a bleeding phenotype.^9^ Treatment with imlifidase (together with rituximab and plasma exchange) was able to decrease the antibodies, allowing the successful transfusion of donor platelets. This case illustrates the potential of creating a window of opportunity for platelet transfusions in a range of settings where alloimmunization occurs. This might even open an option for rare platelet disorders, such as Glanzmann thrombasthenia, where the presence of anti‐HLA and anti‐α2bβ3 antibodies forms a major clinical challenge.

## DOWNSIDE OF IMLIFIDASE

Most clinical experience with imlifidase comes from clinical studies in renal transplantation setting, where it is used for HLA desensitization. Imlifidase has a favorable safety profile, with seven related adverse events in 44 patients described, including five infections and one infusion‐related reaction.^10^ The two serum sickness‐like reactions in iTTP were remarkable; these reactions were seen in none of the other clinical studies, but they led to the premature cessation of the clinical trial in iTTP.

The most important drawback is the immunogenicity of imlifidase itself, where antidrug antibodies develop in almost all individuals. This is not surprising, as *S. pyogenes* is a common infection leading to antibody development in almost all adults.^1^ Imlifidase will boost this antibody response within 1–2 weeks, lasting for an average of 6 months. It is therefore unknown if repeated dosing is possible. From a mechanistic point of view, imlifidase would be able to degrade these IgG antibodies to the same extent as all other IgG antibodies, but for the moment, clinical data on repeated dosing are not available.

Finally, the very high price (around 150.000 euros^11^) will hamper the use of imlifidase for rare, off‐label indications. Currently, the EMA approval is for adult patients waiting for a kidney transplant who are highly sensitized against tissue from the donor and who have a positive crossmatch test against an available kidney from a deceased donor.

In conclusion, imlifidase shows promising results in a broad range of alloimmune hematological conditions. It might be able to create a temporary window of opportunity for treatment in the absence of neutralizing antibodies. Clinical data on hematologic disorders is needed.

## AUTHOR CONTRIBUTIONS

Roger E. G. Schutgens designed and wrote the paper.

## CONFLICT OF INTEREST STATEMENT

The institution of R. E. G. Schutgens has received speaker's fees and/or research grants from Bayer, CSL Behring, Hemab, Novartis, NovoNordisk, Octapharma, Roche, Sobi, and Takeda.

## FUNDING

This research received no funding.

## Data Availability

Data sharing is not applicable to this article as no new data were created or analyzed in this study.
